# Novel efficient genome-wide SNP panels for the conservation of the highly endangered Iberian lynx

**DOI:** 10.1186/s12864-017-3946-5

**Published:** 2017-07-21

**Authors:** Daniel Kleinman-Ruiz, Begoña Martínez-Cruz, Laura Soriano, Maria Lucena-Perez, Fernando Cruz, Beatriz Villanueva, Jesús Fernández, José A. Godoy

**Affiliations:** 1 Departamento de Ecología Integrativa, Estación Biológica de Doñana (EBD-CSIC), Calle Americo Vespucio 26, 41092 Sevilla, Spain; 2 CNAG-CRG, Centre for Genomic Regulation (CRG), Barcelona Institute of Science and Technology (BIST), Baldiri i Reixac 4, 08028 Barcelona, Spain; 3 Departamento de Mejora Genética Animal, INIA, Carretera de la Coruña Km. 7, 28040 Madrid, Spain

**Keywords:** SNPs, STRs, Genetic management, Monitoring, Non-invasive, Genome-wide

## Abstract

**Background:**

The Iberian lynx (*Lynx pardinus*) has been acknowledged as the most endangered felid species in the world. An intense contraction and fragmentation during the twentieth century left less than 100 individuals split in two isolated and genetically eroded populations by 2002. Genetic monitoring and management so far have been based on 36 STRs, but their limited variability and the more complex situation of current populations demand more efficient molecular markers. The recent characterization of the Iberian lynx genome identified more than 1.6 million SNPs, of which 1536 were selected and genotyped in an extended Iberian lynx sample.

**Methods:**

We validated 1492 SNPs and analysed their heterozygosity, Hardy-Weinberg equilibrium, and linkage disequilibrium. We then selected a panel of 343 minimally linked autosomal SNPs from which we extracted subsets optimized for four different typical tasks in conservation applications: individual identification, parentage assignment, relatedness estimation, and admixture classification, and compared their power to currently used STR panels.

**Results:**

We ascribed 21 SNPs to chromosome X based on their segregation patterns, and identified one additional marker that showed significant differentiation between sexes. For all applications considered, panels of autosomal SNPs showed higher power than the currently used STR set with only a very modest increase in the number of markers.

**Conclusions:**

These novel panels of highly informative genome-wide SNPs provide more powerful, efficient, and flexible tools for the genetic management and non-invasive monitoring of Iberian lynx populations. This example highlights an important outcome of whole-genome studies in genetically threatened species.

**Electronic supplementary material:**

The online version of this article (doi:10.1186/s12864-017-3946-5) contains supplementary material, which is available to authorized users.

## Background

Genetic markers provide powerful tools for the study and conservation of biodiversity. They provide valuable insights into population sizes, mating systems, relatedness, population structure, and dispersal rates [1, 2], which can be effectively integrated into management and monitoring programmes of wildlife populations. Such programmes typically involve one or several of the following tasks: (i) individual identification, which allows for the estimation of census sizes, the identification of individual ranges and movements, and some forensic applications [3]; (ii) parentage assignment, which is important in the study of mating systems and reproductive success and contributes to pedigree reconstruction [4]; (iii) relatedness estimation, which in addition to informing on important aspects of species biology, is of highest relevance for the genetic management of populations, as it forms the basis of the commonly used kinship minimization strategy [5]; and (iv) detection of hybridization or admixture, as classification of individuals into specific ancestry categories might prove useful for comparing the fitness consequences of admixture (e.g. [6]). In addition, the genetic information gained can be used for the estimation of effective population size, genetic diversity, gene flow, and to infer demographic changes. Most importantly, all these tasks can be performed on genotypes obtained from non-invasively collected samples, avoiding capture and minimizing risks to animals and people [7].

Microsatellites or short-tandem repeats (STR) have been the markers of choice for the genetic monitoring and management of populations for the last two decades [8, 9], but Single-Nucleotide Polymorphisms (SNPs) have been advocated as superior markers [10, 11]. SNPs are single base nucleotide variants which make up a stable polymorphism in a species [12], and represent the most abundant and widespread source of sequence variation within genomes [13]. Their appeal with respect to STRs also builds on: (i) their lower mutation rates [14], which in turn imply less homoplasy [3], (ii) their lower expected error rates both in genotyping and allele calling (e.g. [15]), and (iii) their automation potential (reviewed in [16]). Finally, and contrary to what happens with STRs, SNP based assays are highly repeatable and easy to standardize across collaborators [17]. At the same time, their biallelic nature often implies lower resolution and statistical power per marker compared to the multiallelic STRs, but this can be easily counterbalanced by increasing the number of SNPs [18].

All in all, SNPs allow for a notorious increase in the power and accuracy of most genetic analyses when available in sufficient numbers. SNP discovery in non-model species, although still a major hurdle, has been greatly facilitated by recent advancements in Next Generation Sequencing (NGS) [19]. Nanoscale genetic analyses on microfluidic platforms [20] have streamlined, reduced costs, and added flexibility to the genotyping assays, minimizing at the same time the DNA quality and quantity requirements of chip-based technologies [17, 21]. Furthermore, the genotyping costs and the effort necessary to achieve the required power can be drastically reduced by selecting the most informative SNP for each particular application. Indeed, high-throughput SNP genotyping has been successfully applied to the non-invasive genetic monitoring of some wild mammal populations based on scat samples [17, 22], and to genetic studies based on museum samples [23].

The Iberian lynx (*Lynx pardinus*) has been highlighted as the most endangered felid species in the world [24]. Seven out of the nine populations extant in the 1980s became extinct by the end of the twentieth century [25], and the remaining two, Doñana (DON) and Andújar (AND), separated by ca. 240 km [26], have been effectively isolated at least since the 1950s (ca. 14 generations) until the start of translocations in 2007 [27]. Several studies have revealed extremely low mitochondrial and STR diversity, record low genome-wide diversity, and excess of potentially deleterious variants, especially in the smallest population (DON), as well as high differentiation between the two populations [28–31]. Most importantly, evidence of inbreeding depression is accumulating [32–35] and the occurrence of an extinction vortex has been suggested for the DON population [30].

Early in the twenty-first century the Iberian lynx dramatic decline prompted the implementation of active conservation strategies with the main funding of three successive European LIFE projects [27]. Conservation actions include (i) the close-up monitoring of individuals, (ii) an ex situ breeding programme that manages a captive population (CAP) resulting from the admixture of the two remnant differentiated genetic pools (DON and AND), (iii) genetic rescue through translocations between these two populations, and (iv) a reintroduction programme aimed at recovering the species’ historical distribution in Spain and Portugal. Population monitoring so far has mostly relied on radio-tracking and camera trapping [36–38], while marker-assisted genetic evaluation and management have been based on 36 STR markers [28, 39]. The remarkable success of these actions has been endorsed by the recent downlisting of the species from “Critically Endangered” to “Endangered” in the 2015 International Union for Conservation of Nature (IUCN) Red List [40], and also by the latest census (2015), which estimates a total of 406 free-living individuals in the Iberian Peninsula across all remnant and reintroduced populations (http://www.iberlince.eu/images/docs/3_InformesLIFE/Informe_Censo_2015.pdf). In spite of this, the species is still in high risk of extinction, and its survival heavily dependent on continued conservation measures.

Given that in recent years many reintroduced populations have been founded by individuals from the admixed CAP population and several translocation events have been carried out between DON and AND, currently all Iberian lynx populations harbour varying degrees of genetic admixture. Such an increasingly complex and interwoven conservation landscape poses new challenges and demands tools as informative and efficient as possible for an integrative management and monitoring of the species.

In this study, we analysed data for ca. 1500 SNPs previously selected from whole genome sequences and previously genotyped in 380 Iberian lynx samples from five populations [31]. The resulting genotypes are analysed here for heterozygosity, Hardy-Weinberg equilibrium (HWE), and linkage disequilibrium (LD). We also identify sex chromosome markers and estimate genotyping error rates. Finally, we rank reliable autosomal SNPs by their informativeness for individual identification, parentage assignment, relatedness, and admixture estimation, and we identify optimal subsets of SNPs for each of these tasks.

## Methods

### SNP selection, samples, and genotyping

Out of >1.6 million SNPs identified through whole genome sequencing of 11 Iberian lynx males [31], a total of 1536 SNPs were selected based on their global Minor Allele Frequency (MAF ≥ 0.4), inter-SNP distance (*d* > 0.6 Mb), and genotyping quality score predicted by the Illumina’s Assay Design Tool algorithm (ADT > 0.9). A total of 380 Iberian lynx blood and muscle samples, including 28 replicates as internal controls, were genotyped for these SNPs using Illumina GoldenGate technology. Genotypes were successfully obtained for 349 samples corresponding to 329 individuals (and 20 replicates).

Markers and samples with more than 20% of missing genotypes were identified with PLINK v1.90b3.36 [41] and discarded from further analyses. To estimate an error rate for the final dataset we first verified replicate genotypes (Additional file 1: Table S1) with PLINK’s Identity By State (IBS) test, and then we tallied discordant genotypes within individual and divided them by the total number of actual pairwise comparisons using a custom R script. Replicate genotypes were discarded from further analyses.

### Pedigree refinement and Mendelian inconsistencies

With the purpose of refining the pedigree and, ultimately, identifying markers departing from autosomal Mendelian inheritance (including X-linked markers), we assessed Mendelian inconsistencies based on known parent-offspring (PO) relationships using the --mendel function in PLINK. Some of these discrepancies allowed us to identify a few misassignments and amend the current pedigree. Additionally, some previously unknown relationships were uncovered by maximum likelihood analyses using ML-RELATE [42] and COLONY [43]. Markers in the refined version of the pedigree that systematically yielded Mendelian incompatibilities between sires and their male offspring were flagged as X-linked markers and treated separately from the main autosomal database unless otherwise specified. Next, we performed the Fisher’s exact test in R [44] to assess differences in allele and genotype frequencies between sexes, which could be indicative of sex-linked inheritance or just genotyping artefacts. Finally, Mendelian inconsistencies in PO dyads and triads in the filtered autosomal database were quantified as a proxy for error rate.

### Basic population genetics statistics

To characterize the genetic pools of DON and AND, we used all pure DON (P_DON_; *N* = 91) and all pure AND (P_AND_; *N* = 165) individuals, considering as such all individuals born in the respective population before their admixture, as well as any wild- or captive-born descendant from two individuals of the same pure ancestry. For each pool, basic summary statistics per marker such as their MAF, observed and expected heterozygosity (H_O_ and H_E_, respectively), and the F_ST_ between the two pools were generated for both the autosomal and the X-linked sets using the *snpStats* R package [45]. A Wilcoxon signed-rank test was performed to compare average H_E_ values between the two pools. The autosomal F_IS_ for each of the two pools was estimated using the R package *hierfstat* [46].

### HWE- and LD-based filtering

As a first step towards selecting a subset of autosomal SNPs for application to conservation, a two-tailed z-test for HWE was performed on each of the two pools of pure individuals with the R package *snpStats*, after discarding monomorphic markers within each pool. For the X-linked set, specific HWE two-sided exact tests were applied using the R package *HardyWeinberg* [47]. Resulting *p*-values were ranked and corrected for multiple testing using the Benjamini-Hochberg (BH) procedure [48].

Next, given that LD between markers in a panel can artificially inflate its power, we defined sample subsets of supposedly unrelated individuals (for P_DON_, *N* = 35; for P_AND_, *N* = 45), we discarded monomorphic markers within each pool, and we used the R package *snpStats* to calculate –for both the autosomal and the X-linked panels– the pairwise *r* ^*2*^ (the squared correlation coefficient between SNP pairs) between all markers. We applied PLINK’s command --indep-pairwise to both the P_DON_ and the P_AND_ autosomal panels, with the *r* ^*2*^ threshold parameter set to a value of 0.5 to prune in one SNP from each pair with *r* ^*2*^ above the threshold. By intersecting the two pruned lists with the R package *VennDiagram* [49], we obtained a set of 343 autosomal SNPs minimally linked in both populations.

### Evaluation of panels for different applications

To optimize marker-assisted monitoring and management, we pursued the identification of the most informative SNPs for individual identification, parentage assignment, relatedness estimation, and ancestry estimation. Based on the allelic frequencies of the 136 individuals from CAP (the main source of individuals for ongoing reintroductions), we estimated statistics relevant to each application (see below). Then, for each statistic, we ranked SNPs and obtained global values for the 343 SNP set as well as for the top 192, 96, 48, 24, and 12 markers. Note that optimal panels should be redesigned for populations with significantly different allelic frequencies. For comparison purposes, the same statistics were also estimated for the panel of 36 STR markers currently used, as well as for a reduced set with the 12 STRs of highest H_E_, using the genotypes of 314 CAP individuals. These STR markers had been selected among those originally developed for other felid species and showed a low overall diversity in the Iberian lynx, with an average number of 3.75 alleles observed per marker (range: 2 – 11) and an average H_O_ of 0.387 (range: 0.003 – 0.774) [28].

i) *Individual identification.* We used the advanced frequency-based analysis module in GENALEX 6.5 [50] to estimate the probability of identity (PID), i.e. the probability that two randomly chosen individuals within a given population have identical genotypes, and the probability of identity among full siblings (PIDs), recommended when related individuals are included in the sample [51]. Global values for each panel were calculated multiplying PID (or PIDs) across loci.
ii) *Parentage assignment*. We also used GENALEX to obtain three probability estimates for parentage exclusion per marker: the probability of exclusion for one putative parent when the genotype of the other parent is known (PE1), the probability of exclusion for one putative parent when the other parent’s genotype is missing (PE2) and the probability of exclusion for the putative parent pair (PE3), as described by Jamieson & Taylor [52]. The corresponding three probabilities of non-exclusion (PnE = 1 - PE) were then calculated for different SNP sets by multiplying PnE across loci.
iii) *Relatedness estimation*. We sorted the SNPs by their informativeness for relatedness index (I_r_), which was obtained using KININFOR [53]. Given that this parameter is not additive over loci, to report global values for each panel we estimated a second measurement of marker informativeness based on the distribution of the likelihood ratios for two candidate relationships, i.e. the power for relationship inference (PWR) [53, 54]. Three common scenarios of candidate relationships analyses were considered: full-sibs (FS) vs. unrelated (UR), FS vs. half-sibs (HS), and HS vs. UR. A suggested prior distribution {1,1,1} and precision level of 0.01 were used in all runs, with the significance level set to 0.05 and an error rate of 10^−5^ for SNPs and 0.015 for STRs.
iv) *Ancestry and admixture estimation*. We evaluated the power of panels through the rate of correct classification of simulated individuals with varying degrees of admixture. First, HYBRIDLAB [55] was used to simulate a population of 100 individuals for each of the following eight ancestry levels: (i) P_DON_, (ii) P_AND_, (iii) offspring of P_DON_ x P_AND_ cross (F_1_), (iv) offspring of F_1_ x F_1_ (F_2_), (v) first backcross DON (Bc_DON_), (vi) first backcross AND (Bc_AND_), (vii) second backcross DON (2Bc_DON_), and (viii) second backcross AND (2Bc_AND_), based on the genotypes for all 343 SNPs and 36 STRs. This programme creates random genotypes for a specified hybrid population based on the allelic frequencies from each of the two hybridizing groups. Next, we used a Bayesian-Markov Chain Monte Carlo (MCMC) method implemented in NEWHYBRIDS [56], to estimate the posterior probability that simulated individuals from each of the eight ancestry levels fall into any of the possible categories. In order to compare the power of ancestry inference of different marker types, set sizes, and ranking criteria, we analysed: (i) the whole 343 SNP set, (ii) subsets of 192, 96, 48, 24, and 12 SNPs with the highest I_r_; (iii) same-sized SNP panels but ranked according to their F_ST_ between P_DON_ and P_AND_; (iv) all 36 STRs; (v) 12 top-ranking STRs according to H_E_; and (vi) 12 STRs of highest F_ST_. Following exploratory runs, a final run of 11,000 iterations (of which the first 1000 were discarded as burn-in), was conducted for every panel using Jeffrey’s priors. Individuals were then assigned to the ancestry category for which the highest posterior probability was obtained, as long as it exceeded 60%. Otherwise they were deemed as “ambiguous” and remained unassigned. Finally, summary matrices with correct, ambiguous, and cross-classification rates were constructed by comparing the true (simulated) ancestry to that inferred using NEWHYBRIDS. Analogously, empirical genotypes in the final dataset were also classified into ancestry categories using the whole and F_ST_-ranked SNP and STR panels.

## Results

### SNP selection, samples and genotyping

In order to identify highly informative SNP panels for genetic monitoring and management, we analysed SNPs which were previously selected from a whole genome variation scan and were genotyped using Illumina GoldenDate technology [31]. Out of 1536 assayed SNPs, we discarded 42 that failed to yield genotypes for any sample, and two additional SNPs that, upon further inspection, proved monomorphic. For the 1492 markers that remained in the dataset (validation rate: 97.1%), the rate of missing genotypes (i.e. missingness) was always lower than 20%. No SNP showed high missingness only in females, as expected for a typical Y-linked marker. Out of the remaining SNPs, 13 were annotated as coding (eight synonymous and five nonsynonymous), and 889 were in regions of shared synteny with domestic cat (*Felis catus*; Additional file 2: Figure S1).

None of the 349 successfully genotyped samples were removed on account of high missingness. When comparing replicate samples to estimate an error rate, we could not find a single discrepancy among 29,857 valid pairwise genotype comparisons (after discarding missing data) among 14 duplicates and three triplicates, which translates into an error rate lower than 3.35*10^−5^.

### Pedigree refinement and Mendelian inconsistencies

To identify markers departing from autosomal Mendelian inheritance, we tested Mendelian segregation based on high-confidence PO relationships. We identified 21 SNPs consistently yielding PO mismatches (ranging from 25 to 72 per SNP across the pedigree). Nearly all these mismatches occurred between sires and their male offspring, indicating that these markers could be X-linked. None of these markers were in confirmed regions of synteny to domestic cat, but they all were within scaffolds that contain other regions with shared synteny to cat chromosome X. Additionally, three out of the remaining 1471 SNPs were syntenic to cat’s X chromosome. The two more distal markers (1,123,473 and 2,370,918) behaved as autosomal, suggesting they are located within the pseudo-autosomal region (3.7 and 4.7 Mb from the tip, respectively); however, the third one (2270811), which is found slightly farther from the chromosome end (6.2 Mb), showed significant allelic and genotypic differences between males and females in both P_DON_ and P_AND_ (Fisher’s exact test with false discovery rate correction for multiple testing; all *p-*values <1*10^−8^; Additional file 1: Tables S2 and S3, respectively).

Tested PO relationships that were identified as incorrect (i.e. were not supported by likelihood analyses) had a minimum of eight Mendelian inconsistencies at these 1471 SNPs, and this was a case in which the discarded parent was a close relative of the true parent. After excluding wrong assignments, a total of eight Mendelian errors remained across 108 dyads and 77 triads of high confidence. These discrepancies involved one dyad and four triads, with two individuals probably accruing all eight errors (Additional file 2: Figure S2). In any case, Mendelian compatibility analyses suggest a low rate of errors, backing up our estimate based on replicate samples.

### Basic population genetics statistics

To characterize the remnant genetic pools of Doñana and Andújar, we obtained basic diversity and differentiation statistics for the 1471 autosomal SNPs and the two pure ancestry samples (P_DON_ and P_AND_; Additional file 1: Table S4). Average values confirm previous observations of lower diversity in P_DON_ (H_E_ = 0.235, σ^2^ = 0.041 vs. H_E_ = 0.398, σ^2^ = 0.012 in P_AND_; *p* = 2.2*10^−16^) and high differentiation between the two pools (weighted F_ST_ = 0.280, σ^2^ = 0.054). For the 21 X-linked markers, we observed slightly lower diversity, but again different diversity between pools (H_E_ = 0.216, σ^2^ = 0.036 in P_DON_; H_E_ = 0.368, σ^2^ = 0.020 in P_AND_; *p* = 0.001) and higher differentiation than for autosomal markers (weighted F_ST_ = 0.309, σ^2^ = 0.113) (Table 1; Additional file 1: Table S5).

**Table 1:** Number of autosomal and X-linked SNPs per ancestry, and average variability and differentiation statistics

Set	Numberof SNPs	Ancestry	Number of monomorphic SNPs	H_E_	H_O_	F_ST (DON-AND)_	F_IS_
Auto-somal	1471	P_DON_	471	0.235	0.240	0.28	−0.013
		P_AND_	5	0.398	0.408		−0.022
X-linked	21	P_DON_	7	0.216	0.236	0.309	-
		P_AND_	0	0.368	0.352		-

### HWE- and LD-based filtering

Next, we performed a z-test for HWE in order to detect and exclude markers departing from random mating expectations. Six autosomal loci were significantly deviating from HWE in each of the two pools after multiple-testing correction; however, no locus was in HW disequilibrium simultaneously in both pools (Additional file 1: Tables S6 and S7). Similarly, no X-linked locus was in significant HW disequilibrium in P_AND_ and P_DON_ (Additional file 1: Tables S8 and S9).

The power of a panel of markers is maximized when all its members segregate independently from each other, i.e. when they are in linkage equilibrium. We thus first estimated LD by calculating the pairwise *r* ^*2*^ values among all markers. Consistent with the high *d* threshold that guided the initial SNP selection, the average *r* ^*2*^ for the full set of autosomal markers was low: 0.029 (σ^2^ = 0.003) for P_DON_ and 0.020 (σ^2^ = 0.001) for P_AND_ (Additional file 1: Tables S10 and S11, respectively). The average *r* ^*2*^ was slightly higher for X-linked markers for both P_DON_ (*r* ^*2*^ = 0.108, σ^2^ = 0.05; Additional file 1: Table S12) and P_AND_ (*r* ^*2*^ = 0.069, σ^2^ = 0.015; Additional file 1: Table S13). Focusing on the autosomal set, the number of pairwise *r* ^*2*^ above 0.5 was negligible: 1169 out of 499,370 valid comparisons in P_DON_, and 831 out of 1,073,840 valid comparisons in P_AND_. Secondly, we pruned out one SNP from each of these pairs with *r* ^*2*^ above 0.5 in any of the two genetic pools. The resulting set of 343 autosomal markers with minimal linkage in both genetic pools was used to evaluate the power of different marker combinations for different applications.

### Evaluation of panels for different applications

#### Individual identification, parentage assignment, and relatedness estimation

To obtain informative panels for these applications, we ranked the 343 minimally linked SNPs according to six statistics (i.e. PID, PIDs, PE1, PE2, PE3, and Ir), estimated based on the allelic frequencies of CAP. We found a high Kendall rank correlation [57] among all six criteria and between each of them and H_E_ (all *τ* > 0.994; Additional file 1: Table S14). All rankings and selected panels based on the first five criteria (i.e. those related to individual identification and parentage exclusion) were exactly the same, which is consistent with their particularly high rank correlation (*τ* > 0.999). Correlations of these with Wang’s I_r_ were slightly lower (*τ* > 0.998), but the selected panels differed in no more than two SNPs between applications – and often in none at all.

**Fig. 1:**
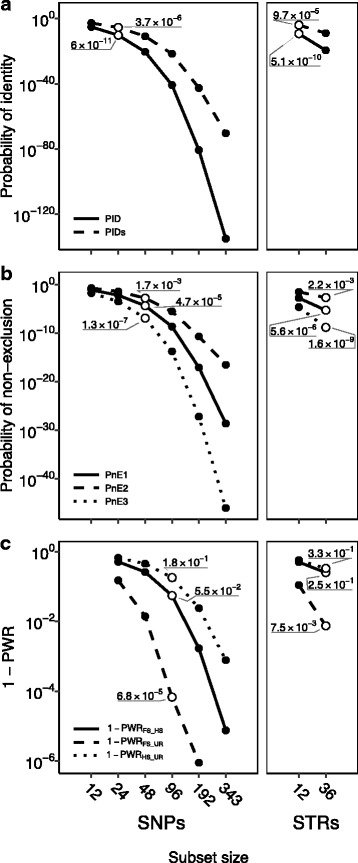
Power of different-sized panels of SNPs and STRs for individual identification (**a**), parentage exclusion (**b**), and relationship discrimination (**c**). White circles indicate the smallest panel informative enough for the respective application, and their power is indicated. 1 – PWR value for the FS vs. UR comparison with 343 SNPs is not shown due to KININFOR’s lack of sensitivity beyond PWR = 0.9999991 (DON: Doñana population; AND: Andújar population; P_DON_: pure DON; P_AND_: pure AND)

Next, we estimated the global values of the aforementioned statistics for the 343 SNP set (Additional file 1: Table S15), for panels with the top ranking 192, 96, 48, 24, and 12 SNPs, for the 36 STR set (Additional file 1: Table S16), and for the 12 STRs of highest H_E_. The 24 SNP panel already surpassed in individual discrimination power (PID = 6.0*10^−11^; PIDs = 3.7*10^−6^) the 12 STR panel currently used for individual identification (PID = 5.1*10^−10^; PIDs = 9.7*10^−5^), and as few as 12 SNPs could still be informative enough to reliably distinguish non extremely inbred or related individuals (Fig. 1a). With regard to parentage exclusion (Fig. 1b), 48 SNPs were enough to approach the already high power of the whole 36 STR set (PnE1 = 4.7*10^−5^; PnE2 = 1.7*10^−3^; PnE3 = 1.3*10^−7^). Finally, the 48 SNP panel’s power to discriminate among alternative relationships was slightly lower than that of 36 STRs, but the 96 SNP set well outperforms it, achieving a power higher than 80% even for the most demanding of the three comparisons (1 - PWR_HS-UR_ = 1.8*10^−1^; Fig. 1c).

#### Admixture and ancestry estimation

Genetic markers can be used to classify individuals in each of the possible ancestry or admixture categories generated following the mixing of the two lynx genetic pools (Additional file 2: Figure S3). For this application, we ranked markers according to their I_r_ and their F_ST_ between P_DON_ and P_AND_. Then, using NEWHYBRIDS, we estimated the power of differently-ranked and -sized panels to classify individuals in ancestry categories as the rate of correct classifications of simulated genotypes generated with HYBRIDLAB (Additional file 1: Table S17).

**Fig. 2:**
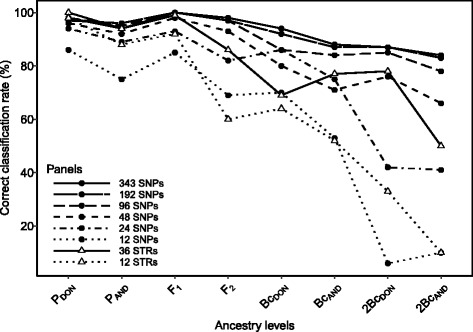
Rate of correct classification of simulated populations with eight distinct degrees of admixture (ancestry levels) for different-sized SNP and STR panels of highest F_ST_ markers (P_DON_: pure DON; P_AND_: pure AND; F_1_: filial generation of P_DON_ x P_AND_ cross; F_2_: offspring of F_1_ x F_1_ cross; Bc_DON_: first backcross DON; Bc_AND_: first backcross AND; 2Bc_AND_: second backcross AND)

Generally speaking, the 343 SNP set was the best performing set. It showed the same power as the set of 36 STRs for the categories of easier discrimination (P_DON_: 98 vs. 100%; P_AND_: 94 vs. 94%; F_1_: 100 vs. 99%) and starkly outperformed it for the remaining categories (F_2_: 98 vs. 86%; Bc_DON_: 94 vs. 69%; Bc_AND_: 88 vs. 77%; 2Bc_DON_: 87 vs. 78%; 2Bc_AND_: 84 vs. 50%). Notwithstanding, the performance of reduced SNP panels of highest I_r_ was not satisfactory (Additional file 1: Table S17). In contrast, panels based on the rank of F_ST_ between P_DON_ and P_AND_ performed comparatively much better. Results for the 96 SNP set show a perfect classification of F_1_ individuals and a highly accurate (>95%) classification of pure and F_2_ individuals, whereas correct assignments were slightly lower for first backcross (Bc_DON_: 86%; Bc_AND_: 84%) and second backcross categories (2Bc_DON_: 85%; 2Bc_AND_: 78%). Only 33 out of the 800 simulated individuals couldn’t be unambiguously assigned to any ancestry level. For smaller panels the classification accuracy fell as expected with size, and particularly so for the less distinguishable backcross and second backcross categories. Overall, the performance of the panel of 48 SNPs was similar to that of the 36 STRs (Fig. 2).

The results of the analyses of empirical data only partially confirmed the patterns observed for simulated data. Larger (SNP) panels showed higher rates of misclassification of pure individuals as second backcrosses than expected from simulations, whereas a better correlation was observed for smaller panels. The sets of 48 SNPs and 36 STRs behaved similarly to each other and to expectations from simulations, with a slightly lower classification of pure DON individuals with SNPs (Additional file 1: Table S18). All F_1_ individuals were perfectly classified across all panels. Unfortunately, admixed categories beyond F_1_ are poorly represented or completely absent from the empirical data, preventing a more thorough comparison of simulated and empirical results.

## Discussion

Our extensive curation and evaluation of a dataset of genotypes for 1536 SNPs allowed us to select maximally informative autosomal SNP panels for individual identification, parentage assignment, relatedness estimation, and admixture estimation. Proposed panels showed higher power than the currently used 36 STR set with only a very modest increase in the number of markers, while adding at the same time the many benefits inherent to SNPs (e.g., their applicability to low quality samples). In combination with flexible genotyping technologies they do provide novel, efficient and cost-effective tools for the demographic and genetic monitoring and management of this highly endangered and actively managed species. Furthermore, we identified 21 X-linked markers that may prove useful for complementing parentage analyses, determining sex, or genetic studies focused on this chromosome.

Genetic monitoring and most forensic applications involve a first step in which genotypes are obtained from new samples and, by checking them against the reference database, are either assigned to already recorded individuals, or identified as previously unsampled individuals. The proposed panel of 24 SNPs has sufficient power to discriminate even between closely related individuals. In a subsequent step in genetic monitoring, novel individuals may be assigned to parents so that a population genealogy is progressively built (e.g. [4]). We found that a panel of only 48 SNPs provides sufficient power to assign parentage with high confidence, even in the most demanding situation where none of the parents are known. It must be noted, though, that more markers (e.g. 96 SNPs) could be needed in specific scenarios with multiple, closely related putative sires [58], as observed in a previous study on reproduction patterns in the DON population (Lucena-Perez et al. submitted).

One important feature of genetic monitoring is that it can be based on non-invasively collected samples, such as faeces, hairs or feathers [59], and here SNPs offer important advantages over STRs [3, 60]. Degraded DNA in low quality samples is prone to low rates of amplification and high rates of genotyping errors, including false alleles and allelic dropout [61]. Reported error rates for STR markers vary greatly among studies, ranging from 0% to above 30% when considering just allelic dropout (reviewed in [62]), thus imposing the need for multiple genotyping replicates of the same extract [63]. However, degraded DNA can be typed more efficiently and accurately for SNPs due to their considerably shorter target DNA sequence (~50-70 bp) compared to STRs’ (80–300 bp) [3]. Indeed, the few existing studies on SNP genotyping of non-invasively collected wildlife samples report low rates of genotyping failure and errors, e.g. <10% and ~1%, respectively, in wolves [17], and 0.36 and 0.038% in bears [22], eliminating the need for costly and laborious systematic replicates.

The other very important use of genetic markers in conservation is genetic management, which is typically based on a kinship minimization strategy [5]. This strategy is presently being applied to the management of the captive Iberian lynx population using STR-based estimations of relatedness among founders in combination with the completely known genealogy [39]. However, the currently used 36 STR set in fact provides limited information on relatedness, since it correctly discriminates <80% –a threshold used by Wang [53]– of FS from HS dyads at a significance level of *p* < 0.05. On the contrary, 96 SNPs provide enough power to correctly discriminate >80% of HS from UR dyads, which is the most demanding of the three tested comparisons. Still, even our whole 343 SNP panel does not provide enough density to completely replace genealogy in kinship minimization strategies, which has been estimated in 3N_e_ SNPs/Morgan [64]. On the other hand, even when quite complete genealogies are available, genetic markers can be used to estimate the unknown relatedness among the founders. Furthermore, genetic markers do estimate the realized –rather than the expected– kinship or inbreeding (which can be more useful measures for most applications) for the whole genome or for specific regions [64, 65].

The increasingly complex situation of Iberian lynx conservation demands the extension of the genetic management to the remnant and reintroduced populations, ideally within a single integrative genetic management programme for the species. Relatedness estimation based on the novel SNP panels presented here will become most critical for this objective, since with the exception of DON (Lucena-Pérez et al. submitted), other populations lack reliable and reasonably complete genealogies.

The fact that PID, PIDs, PnE, and I_r_ share a nearly identical ranking of markers means that panels designed for a less demanding purpose (e.g. individual identification) are nested within those for more demanding applications (e.g. parentage assignment). This enables a module workflow that grants great flexibility to genetic monitoring and management of Iberian lynx populations. For example, non-invasively collected samples would be assigned to specific individuals based on the 12–24 SNP panels. The novel unique genotypes could then be assigned to parents and incorporated to the pedigree after genotyping one of its replicate samples for the next most informative 24–72 SNPs. Finally, individual lynx that are candidates for being translocated or incorporated into captivity could be additionally typed for the remaining SNPs for relatedness estimation.

We also considered admixture estimation as a potential application of the novel SNP markers, since the two highly differentiated genetic pools have recently been admixed in captivity and in the wild through translocations. Differentiating individuals by ancestry might be most relevant for the assessment of the fitness consequences of admixture, i.e. for the evaluation of genetic rescue or outbreeding depression (e.g. [6]). Since most of the markers of highest H_E_ are poorly differentiated between the two pure ancestries, admixture tests would require their own particular sets of ancestry informative markers, with little overlap with those selected for other applications. For this purpose, using loci with different alleles fixed in each population would be optimal, but these were excluded by the MAF requirement in the initial selection. Fortunately, a sufficient number of highly differentiated SNPs were available to provide enough power for ancestry classification. Simulated individuals of P_DON_, P_AND_, F_1_, and F_2_ categories are reasonably well classified with the 48 highest F_ST_ SNP set and the 36 STR set, whereas less differentiated categories require at least 96 SNPs.

The observed accuracy of ancestry classification of empirical genotypes with SNP panels became lower than that inferred from simulated genotypes as panel size increased. This is probably due to the additional LD derived from the relatively high relatedness structure and, especially, the recent admixture in current Iberian lynx populations (admixture linkage disequilibrium) [66]. This effect gains relevance as the density of markers increases, and is expected to dilute out with successive generations of random mating following admixture [67]. This should also result in the overestimation of power of individual discrimination, parentage assignments, and relationship inference, which assume markers combined in the same panel are completely unlinked. However, individual identification and parentage assignment shouldn’t be much affected by this, since i) the proposed panels are small, and ii) the threshold of estimated power that we chose for these applications is well above the minimum required, an advice that could be extended to similar studies in other species to account for any possible deviation of theoretical or simulation assumptions. Finally, even if not completely realistic, simulations and theoretical power estimations are still useful for the comparison among marker types and panel sizes.

Our observed SNPs to STRs ratio (e.g. around 1.3 for individual identification) is at the lower end of or even below the range of 1.7 to 5.56 SNPs per STR reported in humans (see [18] and the references therein). The high performance of SNPs when compared to STRs found here can be explained by the restrictive selection of the most informative among the more than 1.6 million SNPs found in the analyses of 11 Iberian lynx genomes [31]. In contrast, the 36 STRs were selected among far fewer markers, which were also originally developed for other felid species: 25 out of ~250 from domestic cat, seven out of eight from bobcat (*Lynx rufus*), and four out of six from Canadian lynx (*Lynx canadensis*) [28]. This more limited search and the ascertainment bias associated to the heterologous nature of these markers have limited the variability of the selected STRs, to the point where some of the worst performing STRs have lower H_E_ than any of the selected SNPs.

These results highlight the importance of the variant discovery and screening phases. Once considered a major limitation of SNP adoption, NGS approaches have greatly facilitated these tasks in non-model organisms, especially when applied to reduced genome subsets or transcriptomes [68]. Still, such screening effort will be especially challenging for genetically eroded species, requiring a more extensive sampling of the genome (as exemplified here) or yielding larger panels for any given power than in genetically diverse species.

The decision on how many markers to use for the most demanding applications (e.g. relatedness estimation) will mostly rely on cost-budget considerations, and these heavily depend on the genotyping technology used. The excessive cost of chip-based technologies –but also their limited flexibility and high input sample requirement– have deterred the application of non-invasive SNP-based approaches in wildlife. On the other hand, technologies based on fluorescent detection provide the advantage of a single PCR reaction, thus avoiding multiple manipulation steps [69], and when implemented with nanofluidic systems, the sample, reagent, and labour requirements are substantially reduced. For example, the Fluidigm system enables flexible and high-throughput SNP genotyping through the use of dynamic arrays with Integrated Fluidic Circuits (IFCs) of different sizes, such as 48.48 (48 samples against 48 markers per run) or 192.24 (192 samples against 24 markers). The philosophy behind this technology fits well the needs of a conservation programme, where samples may not accrue at a uniform pace, and questions requiring different SNP sets might need to be addressed as soon as they arise. Moreover, hands-on laboratory costs with the Fluidigm system are substantially cheaper than for STR genotyping, and initial investments in synthesis of assay probes are now similar – but SNP probes can be applied to a much larger number of assays [17]. Thus, in the long term, the Fluidigm system stands as an efficient and cost-effective option for continued monitoring of wildlife.

## Conclusions

The Iberian lynx example highlights one valuable practical outcome of whole-genome studies in heavily eroded species, i.e. the identification of scarce highly informative markers that will most likely be missed in smaller scale screenings. Indeed, highly efficient and cost-effective marker panels for genetic monitoring and management are likely the most immediate and feasible contribution of genomics to endangered species conservation.

The Iberian lynx is a quite extreme example of genetically eroded and intensively managed species, with ongoing conservation actions including captive breeding, translocations and reintroductions. The long-term success of such actions, and ultimately the viability of the species, will largely depend on the implementation of sound, efficient, and science-driven monitoring and management programmes. As the novel SNP markers presented here provide higher power, efficiency, and flexibility than currently used STRs, they can make a substantial contribution toward this goal by guiding the comprehensive genetic management of captive, remnant and reintroduced populations and, in combination with non-invasive sampling, by complementing –and eventually substituting– monitoring programmes currently based almost exclusively on radiotracking and camera-trapping.

## Additional files

Additional file 1: Table S1. Genotypes of replicate samples. **Table S2.** Results of the Fisher’s exact test on differences in allele and genotype frequencies between sexes for the P_DON_ pool. **Table S3.** Results of the Fisher’s exact test on differences in allele and genotype frequencies between sexes for the P_AND_ pool. **Table S4.** Basic population genetics statistics per autosomal marker for both genetic pools. **Table S5.** Basic population genetics statistics per X-linked marker for both genetic pools. **Table S6.** Results for HWE analysis per autosomal marker for the P_DON_ pool. **Table S7.** Results for HWE analysis per autosomal marker for the P_AND_ pool. **Table S8.** Results for HWE analysis per X-linked marker for the P_DON_ pool. **Table S9.** Results for HWE analysis per X-linked marker for the P_AND_ pool. **Table S10.** Hemimatrix of pairwise *r* ^*2*^ values between autosomal markers for the P_DON_ pool. **Table S11**. Hemimatrix of pairwise *r* ^*2*^ values between autosomal markers for the P_AND_ pool. **Table S12.** Hemimatrix of pairwise *r* ^*2*^ values between X-linked markers for the P_DON_ pool. **Table S13.** Hemimatrix of pairwise *r* ^*2*^ values between X-linked markers for the P_AND_ pool. **Table S14.** Hemimatrix of Kendall rank correlation *τ* values between H_E_ and statistics selected to rank markers. **Table S15.** Informativeness and rankings for individual identification, parentage assignment, and relatedness estimation of SNPs in the 343 SNP panel. **Table S16.** Informativeness and rankings for individual identification, parentage assignment, and relatedness estimation of STRs in the 36 STR panel. **Table S17**. Summary matrices with NEWHYBRIDS’ correct, ambiguous, and cross-classification rates of simulated individuals in eight ancestry levels, for differently-sized and -ranked SNP and STR panels. **Table S18**. Summary matrices with NEWHYBRIDS’ correct, ambiguous, and cross-classification values (and percentages) of empirical individuals in eight ancestry levels, for different-sized SNP and STR panels. (XLSX 33994 kb)

Additional file 2: Figure S1. Location of syntenic SNPs in the domestic cat’s chromosomes. **Figure S2.** Family trees accruing all eight Mendelian errors concerning SNP 1317372 (A); SNP 334649 (B); SNP 619378 (C); SNP 2119932 (D); and SNP 2057563 (E). Dark background: allele A; light background: allele B; white background: unknown allele. **Figure S3.** Ancestry of Iberian lynx individuals in each of the two differentiated genetic pools, as inferred from the analysis of 1,471 autosomal SNPs in STRUCTURE under the assumption of two genetic clusters (K = 2). Each individual is represented by a vertical line with the height of each colour representing the estimated fraction of their genome belonging to each cluster. The black vertical lines delimit groups of individuals of different admixture categories, know *a priori* from their provenance or recent genealogy (P_DON_: pure DON; P_AND_: pure AND; F_1_: filial generation of P_DON_ x P_AND_ cross; F_2_: offspring of F_1_ x F_1_ cross; Bc_DON_: first backcross DON; Bc_AND_: first backcross AND; 2Bc_AND_: second backcross AND). Ten runs of 100,000 MCMC iterations (of which the first 10,000 were discarded as burn-in) were performed in STRUCTURE v2.3.4 with highly concordant results. A representative run is depicted here. (PDF 380 kb)

## Abbreviations

2Bc_AND_: Second backcross to Andújar
2Bc_DON_: Second backcross to Doñana
ADT: Illumina’s assay design tool algorithm
AND: Andújar population of Iberian lynx
Bc_AND_: First backcross to Andújar
Bc_DON_: First backcross to Doñana
BH: Benjamini-Hochberg multiple testing correction
CAP: Captive population of Iberian lynx
DNA: Deoxyribonucleic acid
DON: Doñana population of Iberian lynx
F_1_: Offspring of P_DON_ x P_AND_ cross
F_2_: Offspring of F_1_ x F_1_
FS: Full-siblings relationship
HS: Half-siblings relationship
HW: Hardy-Weinberg
HWE: Hardy-Weinberg equilibrium
IBS: Identity by state
IFC: Integrated fluidic circuit
I_r_: Informativeness for relatedness
IUCN: International union for conservation of nature
LD: Linkage disequilibrium
MAF: Minor allele frequency
MCMC: Markov Chain Monte Carlo
NGS: Next generation sequencing
P_AND_: Pure AND individuals
PCR: Polymerase chain reaction
P_DON_: Pure DON individuals
PE: Probability of exclusion
PE1: Probability of exclusion for one putative parent when the genotype of the other parent is known
PE2: Probability of exclusion for one putative parent when the other parent’s genotype is missing
PE3: Probability of exclusion for the putative parent pair
PID: Probability of identity
PIDs: Probability of identity among full siblings
PnE: Probability of non exclusion
PO: Parent-offspring relationship
PWR: Power for relationship inference
SNP: Single-nucleotide polymorphism
STR: Short-tandem repeat (i.e. microsatellite)
UR: Unrelated individuals

## References

[1] Allendorf FW, Hohenlohe PA, Luikart G (2010). Genomics and the future of conservation genetics. Nat Rev Genet Nature Publishing Group.

[2] Frankham R, Ballou JD, Briscoe DA (2010). Introduction to conservation genetics.

[3] Morin PA, Luikart G, Wayne RK (2004). The SNP workshop group. SNPs in ecology, evolution and conservation. Trends Ecol Evol.

[4] De Barba M, Waits LP, Garton EO, Genovesi P, Randi E, Mustoni A (2010). The power of genetic monitoring for studying demography, ecology and genetics of a reintroduced brown bear population. Mol Ecol.

[5] Caballero A, Toro MA (2000). Interrelations between effective population size and other pedigree tools for the management of conserved populations. Genet Res.

[6] Miller JM, Poissant J, Hogg JT, Coltman DW (2012). Genomic consequences of genetic rescue in an insular population of bighorn sheep (Ovis Canadensis). Mol Ecol.

[7] Goossens B, Bruford MW, Bertorelle G, Bruford MW, Hauffe HC, Rizzoli A, Vernesi C (2009). Non-invasive genetic analysis in conservation. Popul. Genet. Anim. Conserv.

[8] Schlötterer C (2004). Opinion: the evolution of molecular markers — just a matter of fashion?. Nat. Rev. Genet..

[9] Selkoe KA, Toonen RJ. Microsatellites for ecologists: a practical guide to using and evaluating microsatellite markers. Ecol Lett. 2006;615–29. 10.1111/j.1461-0248.2006.00889.x 16643306

[10] Belfiore NM, Hoffman FG, Baker RJ, Dewoody JA (2003). The use of nuclear and mitochondrial single nucleotide polymorphisms to identify cryptic species. Mol Ecol.

[11] Bensch S, Akesson S, Irwin DE (2002). The use of AFLP to find an informative SNP: genetic differences across a migratory divide in willow warblers. Mol Ecol.

[12] Brookes AJ. The essence of SNPs. Gene. 1999;177–86. 10.1016/s0378-1119(99)00219-x 10395891

[13] Brumfield RT, Beerli P, Nickerson DA, Edwards S V. The utility of single nucleotide polymorphisms in inferences of population history. Trends Ecol Evol. 2003;249–56.

[14] Estoup A, Jarne P, Cornuet JM. Homoplasy and mutation model at microsatellite loci and their consequences for population genetics analysis. Mol Ecol. 2002;1591–604. 10.1046/j.1365-294x.2002.01576.x 12207711

[15] Quillery E, Quenez O, Peterlongo P, Plantard O (2014). Development of genomic resources for the tick Ixodes ricinus : isolation and characterization of single nucleotide polymorphisms. Mol Ecol Resour.

[16] Olsen MT, Volny VH, Bérubé M, Dietz R, Lydersen C, Kovacs KM (2011). A simple route to single-nucleotide polymorphisms in a nonmodel species: identification and characterization of SNPs in the Artic ringed seal (Pusa Hispida Hispida). Mol Ecol Resour.

[17] Kraus RHS, VonHoldt B, Cocchiararo B, Harms V, Bayerl H, Kühn R (2015). A single-nucleotide polymorphism-based approach for rapid and cost-effective genetic wolf monitoring in Europe based on noninvasively collected samples. Mol Ecol Resour.

[18] Gärke C, Ytournel F, Bed’Hom B, Gut I, Lathrop M, Weigend S (2012). Comparison of SNPs and microsatellites for assessing the genetic structure of chicken populations. Anim Genet.

[19] Davey JW, Hohenlohe PA, Etter PD, Boone JQ, Catchen JM, Blaxter ML (2011). Genome-wide genetic marker discovery and genotyping using next-generation sequencing. Nat Rev Genet.

[20] Senapati S, Mahon AR, Gordon J, Nowak C, Sengupta S, Powell THQ, et al. Rapid on-chip genetic detection microfluidic platform for real world applications. Biomicrofluidics. 2009;3. 10.1063/1.3127142 PMC2717575 19693342

[21] Bhat S, Polanowski AM, Double MC, Jarman SN, Emslie KR (2012). The effect of input DNA copy number on genotype call and characterising SNP markers in the humpback whale genome using a nanofluidic array. PLoS One.

[22] Norman AJ, Spong G (2015). Single nucleotide polymorphism-based dispersal estimates using noninvasive sampling. Ecol Evol.

[23] Spitzer R, Norman AJ, Schneider M, Spong G (2016). Estimating population size using single-nucleotide polymorphism-based pedigree data. Ecol Evol.

[24] Nowell K, Jackson P (1996). Wild cats: status survey and conservation action plan, International Union for Conservation of nature (IUCN).

[25] Rodríguez A, Delibes M (2004). Patterns and causes of non-natural mortality in the Iberian lynx during a 40-year period of range contraction. Biol Conserv.

[26] Guzmán JN, García FJ, Garrote G, Pérez de Ayala R, Iglesias C (2004). El lince ibérico (Lynx pardinus) en España y Portugal. Censo diagnóstico de sus poblaciones.

[27] Simón MA, Gil-Sánchez JM, Ruiz G, Garrote G, Mccain EB, Fernández L, et al. Reverse of the decline of the Endangered Iberian lynx. Conserv Biol. 2012;731–6. 10.1111/j.1523-1739.2012.01871.x 22734818

[28] Casas-Marce M, Soriano L, López-Bao JV, Godoy JA (2013). Genetics at the verge of extinction: insights from the Iberian lynx. Mol Ecol.

[29] Johnson WE, Godoy JA, Palomares F, Delibes M, Fernandes M, Revilla E (2004). Phylogenetic and phylogeographic analysis of Iberian lynx populations. J Hered.

[30] Palomares F, Godoy JA, López-Bao JV, Rodríguez A, Roques S, Casas-Marce M (2012). Possible extinction vortex for a population of Iberian lynx on the verge of extirpation. Conserv Biol.

[31] Abascal F, Corvelo A, Cruz F, Villanueva-Cañas JL, Vlasova A, Marcet-Houben M (2016). Extreme genomic erosion after recurrent demographic bottlenecks in the highly endangered Iberian lynx. Genome Biol Genome Biology.

[32] Jimenez MA, Sanchez B, Perez Alenza MD, Garcia P, Lopez JV, Rodriguez A (2008). Membranous glomerulonephritis in the Iberian lynx (Lynx Pardinus). Vet Immunol Immunopathol.

[33] Meli ML, Cattori V, Martínez F, López G, Vargas A, Palomares F (2010). Feline leukemia virus infection: a threat for the survival of the critically endangered Iberian lynx (Lynx Pardinus). Vet Immunol Immunopathol.

[34] Peña L, Garcia P, Jiménez MA, Benito A, Alenza MDP, Sánchez B (2006). Histopathological and immunohistochemical findings in lymphoid tissues of the endangered Iberian lynx (Lynx Pardinus). Comp Immunol Microbiol Infect Dis.

[35] Ruiz-López MJ, Gañán N, Godoy JA, Del Olmo A, Garde J, Espeso G (2012). Heterozygosity-fitness correlations and inbreeding depression in two critically endangered mammals. Conserv Biol.

[36] Garrote G, de Ayala RP, Pereira P, Robles F, Guzman N, García FJ (2011). Estimation of the Iberian lynx (Lynx Pardinus) population in the Doñana area, SW Spain, using capture-recapture analysis of camera-trapping data. Eur J Wildl Res.

[37] Garrote G, de Ayala RP, Tellería JL (2014). A comparison of scat counts and camera-trapping as means of assessing Iberian lynx abundance. Eur J Wildl Res.

[38] Gil-Sánchez JM, Moral M, Bueno J, Rodríguez-Siles J, Lillo S, Pérez J (2011). The use of camera trapping for estimating Iberian lynx (Lynx Pardinus) home ranges. Eur J Wildl Res.

[39] Godoy JA, Casas-Marcé M, Fernández J, Vargas A, Breitenmoser C, Breitenmoser U (2009). Genetic issues in the implementation of the Iberian lynx ex situ conservation programme. Iber. Lynx ex situ Conserv. An Interdiscip. Approach.

[40] Rodríguez A, Calzada J. *Lynx pardinus*. The IUCN Red List of Threatened Species [Internet]. Version 20. IUCN Red List Threat. Species. IUCN; 2015.

[41] Purcell S, Neale B, Todd-Brown K, Thomas L, Ferreira MAR, Bender D (2007). PLINK: a tool set for whole-genome association and population-based linkage analyses. Am J Hum Genet.

[42] Kalinowski ST, Wagner AP, Taper ML (2006). ML-RELATE: a computer program for maximum likelihood estimation of relatedness and relationship. Mol Ecol Notes.

[43] Jones OR, Wang J (2010). COLONY: a program for parentage and sibship inference from multilocus genotype data. Mol Ecol Resour.

[44] The R Core Team (2015). R: A language and environment for statistical computing.

[45] Clayton D (2015). snpStats: SnpMatrix and XSnpMatrix classes and methods. R package version 1.20.0.

[46] Goudet J, Jombart T (2015). Hierfstat: estimation and tests of hierarchical F-statistics. R package version 0.04-22.

[47] Graffelman J (2015). Exploring diallelic genetic markers: the HardyWeinberg package. J Stat Softw.

[48] Benjamini Y, Hochberg Y (1995). Controlling the false discovery rate – a practical and powerful approach to multiple testing. J R Stat Soc B.

[49] Chen H, Boutros PC (2011). VennDiagram: a package for the generation of highly-customizable Venn and Euler diagrams in R. BMC Bioinformatics.

[50] Peakall R, Smouse P (2012). GenAlEx 6.5: genetic analysis in excel. Population genetic software for teaching and research – an update. Bioinformatics.

[51] Waits LP, Luikart G, Taberlet P (2001). Estimating the probability of identity among genotypes in natural populations: cautions and guidelines. Mol Ecol.

[52] Jamieson A, Taylor SC (1997). Comparisons of three probability formulae for parentage exclusion. Anim Genet.

[53] Wang J (2006). Informativeness of genetic markers for pairwise relationship and relatedness inference. Theor Popul Biol.

[54] Goodnight KF, Queller DC (1999). Computer software for performing likelihood test of pedigree relationships using genetic markers. Mol Ecol.

[55] Nielsen EE, Bach LA, Kotlicki P (2006). HYBRIDLAB (version 1.0): a program for generating simulated hybrids from population samples. Mol Ecol Notes.

[56] Anderson EC, Thompson EA (2002). A model-based method for identifying species hybrids using multilocus genetic data. Genetics.

[57] Kendall MG (1938). A new measure of rank correlation. Biometrika.

[58] Fernández ME, Goszczynski DE, Lirón JP, Villegas-Castagnasso EE, Carino MH, Ripoli MV (2013). Comparison of the effectiveness of microsatellites and SNP panels for genetic identification, traceability and assessment of parentage in an inbred Angus herd. Genet Mol Biol.

[59] Schwartz M, Luikart G, Waples R (2007). Genetic monitoring as a promising tool for conservation and management. Trends Ecol Evol.

[60] Seddon JM, Parker HG, Ostrander EA, Ellegren H (2005). SNPs in ecological and conservation studies: a test in the Scandinavian wolf population. Mol Ecol.

[61] Bonin A, Bellemain E, Eidesen PB, Pompanon F, Brochmann C, Taberlet P. How to track and assess genotyping errors in population genetics studies. Mol Ecol. 2004;3261–73. 10.1111/j.1365-294X.2004.02346.x 15487987

[62] Broquet T, Ménard N, Petit E (2007). Noninvasive population genetics: a review of sample source, diet, fragment length and microsatellite motif effects on amplification success and genotyping error rates. Conserv Genet.

[63] Navidi W, Arnheim N, Waterman MS (1992). A multiple-tubes approach for accurate genotyping of very small DNA samples by using PCR – statistical considerations. Am J Hum Genet.

[64] Gómez-Romano F, Villanueva B, de Cara MA, Fernández J (2013). Maintaining genetic diversity using molecular coancestry: the effect of marker density and effective population size. Genet Sel Evol.

[65] Kardos M, Luikart G, Allendorf FW (2015). Measuring individual inbreeding in the age of genomics: marker-based measures are better than pedigrees. Heredity (Edinb).

[66] Loh PR, Lipson M, Patterson N, Moorjani P, Pickrell JK, Reich D (2013). Inferring admixture histories of human populations using linkage disequilibrium. Genetics.

[67] Pfaff CL, Parra EJ, Bonilla C, Hiester K, McKeigue PM, Kamboh MI (2001). Population structure in admixed populations: effect of admixture dynamics on the pattern of linkage disequilibrium. Am J Hum Genet.

[68] Kumar S, Banks TW, Cloutier S. SNP discovery through next-generation sequencing and its applications. Int J Plant Genomics. 2012;2012. 10.1155/2012/831460 PMC3512287 23227038

[69] Fabbri E, Caniglia R, Mucci N, Thomsen HP, Krag K, Pertoldi C (2012). Comparison of single nucleotide polymorphisms and microsatellites in non-invasive genetic monitoring of a wolf population. Arch Biol Sci.

