# Supplementary material for: Novel efficient genome-wide SNP panels for the conservation of the highly endangered Iberian lynx
Source: BMC Genomics. 2017 Jul 21;18:556. doi: 10.1186/s12864-017-3946-5 (PMC5522595; doi:10.1186/s12864-017-3946-5)

Figure S1. Location of syntenic SNPs in the domestic cat's chromosomes.

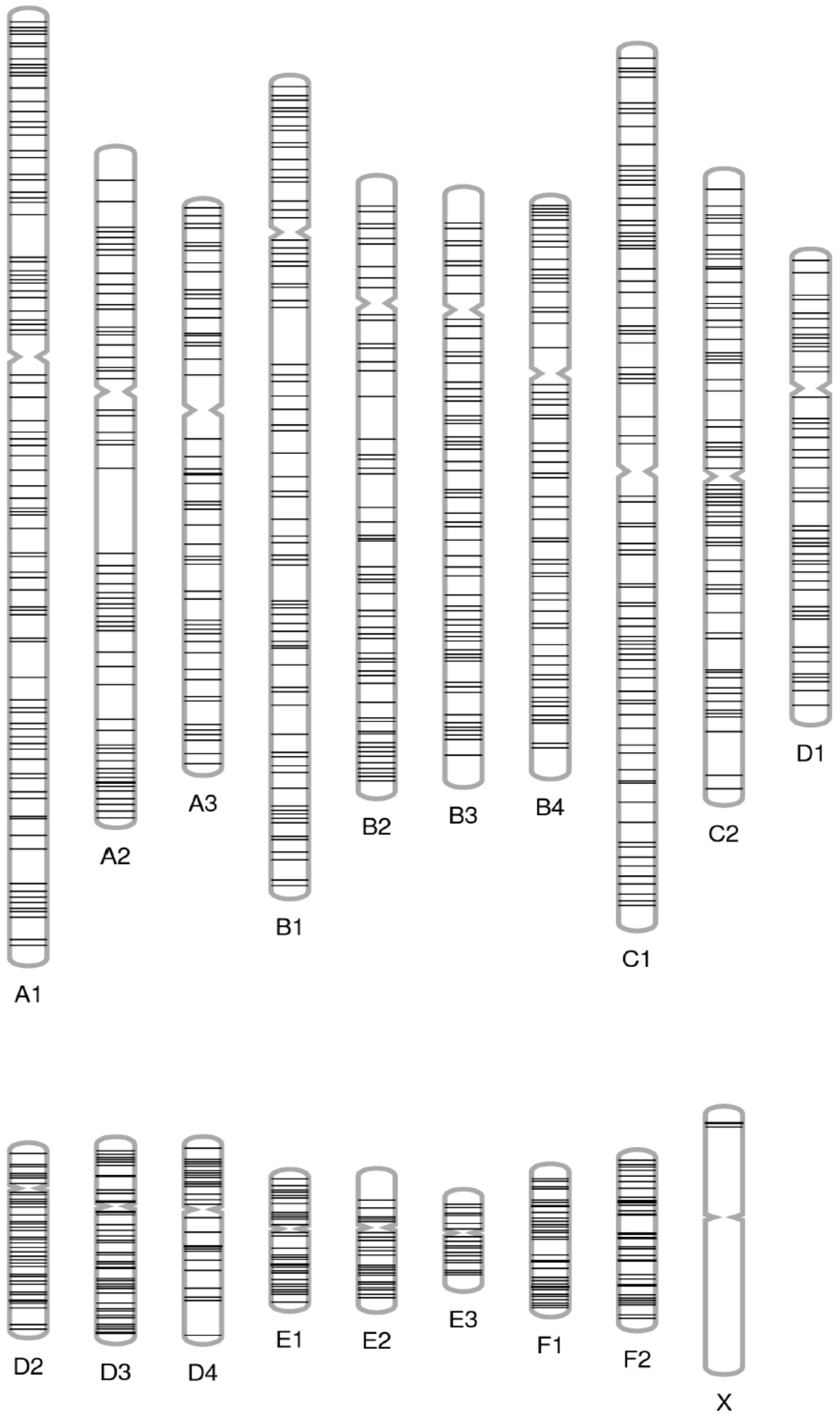

Figure S2. Family trees accruing all eight Mendelian errors concerning SNP 1317372 (A); SNP 334649 (B); SNP 619378 (C); SNP 2119932 (D); and SNP 2057563 (E). Dark background: allele A; light background: allele B; white background: unknown allele.

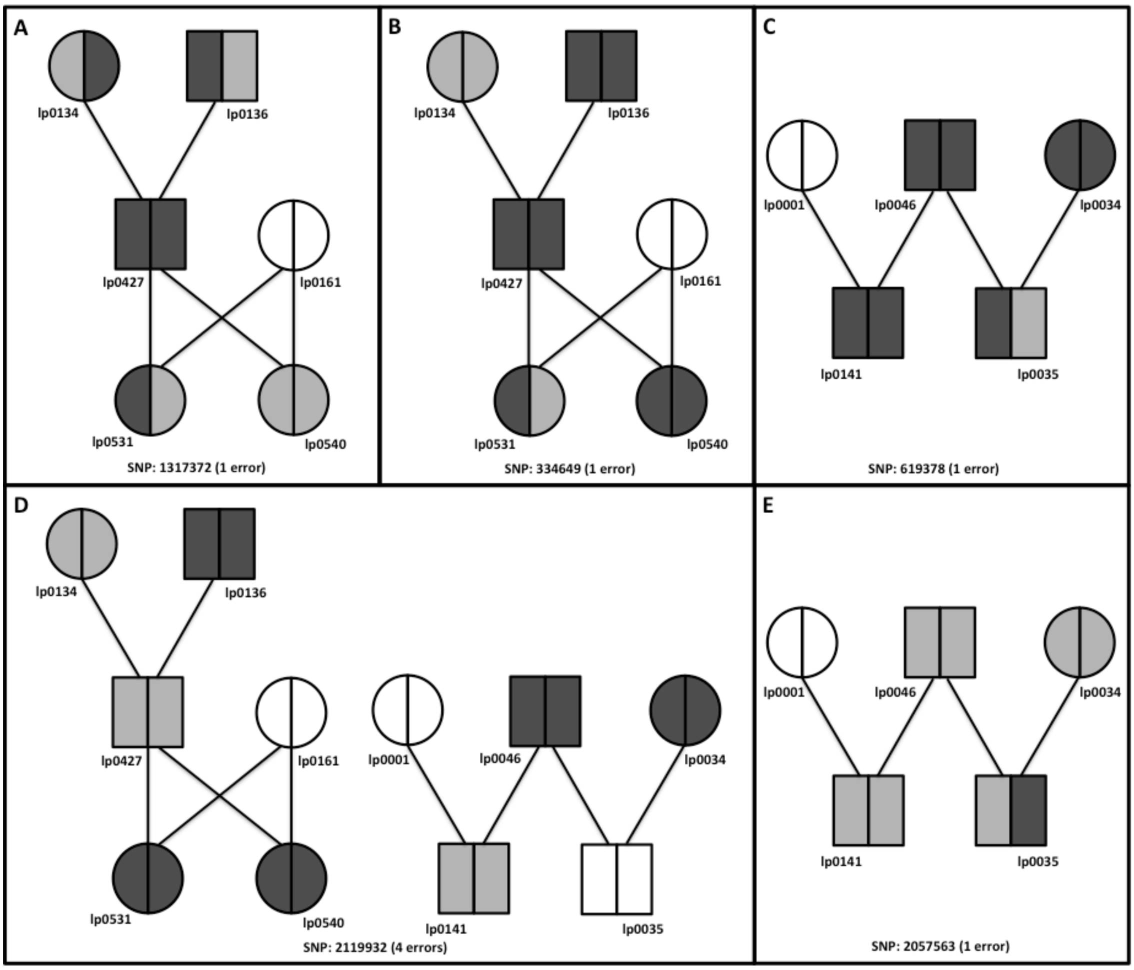

Figure S3. Ancestry of Iberian lynx individuals in each of the two differentiated genetic pools, as inferred from the analysis of 1,471 autosomal SNPs in STRUCTURE under the assumption of two genetic clusters (K = 2). Each individual is represented by a vertical line with the height of each colour representing the estimated fraction of their genome belonging to each cluster. The black vertical lines delimit groups of individuals of different admixture categories, know *a priori* from their provenance or recent genealogy (P_{DON}: pure DON; P_{AND}: pure AND; F_1: filial generation of P_{DON} x P_{AND} cross; F_2: offspring of F_1 x F_1 cross; Bc_{DON}: first backcross DON; Bc_{AND}: first backcross AND; $2Bc_{AND}$: second backcross AND). Ten runs of 100,000 MCMC iterations (of which the first 10,000 were discarded as burn-in) were performed in STRUCTURE v2.3.4 with highly concordant results. A representative run is depicted here.

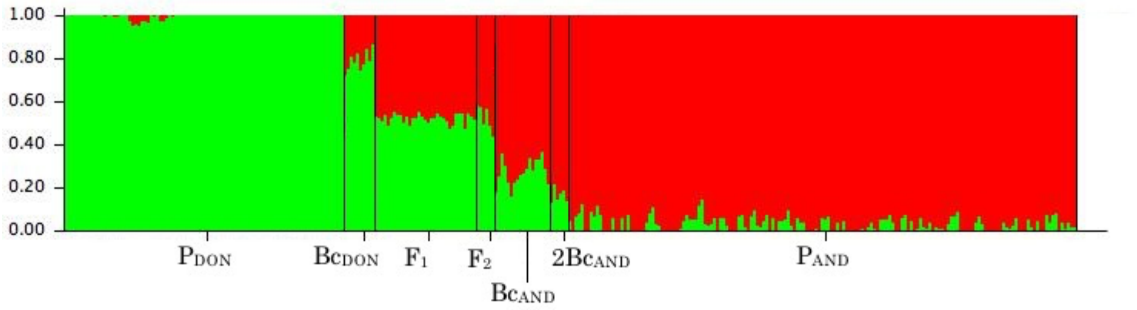

Supplement: Supplementary file 2 — Location of syntenic SNPs in the domestic cat’s chromosomes. Figure S2. Family trees accruing all eight Mendelian errors concerning SNP 1317372 (A); SNP 334649 (B); SNP 619378 (C); SNP 2119932 (D); and SNP 2057563 (E). Dark background: allele A; light background: allele B; white background: unknown allele. Figure S3. Ancestry of Iberian lynx individuals in each of the two differentiated genetic pools, as inferred from the analysis of 1,471 autosomal SNPs in STRUCTURE under the assumption of two genetic clusters (K = 2). Each individual is represented by a vertical line with the height of each colour representing the estimated fraction of their genome belonging to each cluster. The black vertical lines delimit groups of individuals of different admixture categories, know a priori from their provenance or recent genealogy (PDON: pure DON; PAND: pure AND; F1: filial generation of PDON x PAND cross; F2: offspring of F1 x F1 cross; BcDON: first backcross DON; BcAND: first backcross AND; 2BcAND: second backcross AND). Ten runs of 100,000 MCMC iterations (of which the first 10,000 were discarded as burn-in) were performed in STRUCTURE v2.3.4 with highly concordant results. A representative run is depicted here. (PDF 380 kb) [file 12864_2017_3946_MOESM2_ESM.pdf]
